# Measurement of image rotation angle in CT for radiotherapy treatment planning

**DOI:** 10.1120/jacmp.v17i4.6203

**Published:** 2016-07-08

**Authors:** Witold Skrzynski, Wioletta Ślusarczyk‐Kacprzyk

**Affiliations:** ^1^ Department of Medical Physics Maria Sklodowska‐Curie Memorial Cancer Center and Institute of Oncology Warsaw Poland

**Keywords:** CT scanner, image rotation angle, radiation therapy

## Abstract

The geometric accuracy of CT images is essential when they are used for planning radiation therapy. In this study, a method of quantitative testing of image rotation in CT is presented, based on the automated analysis of an image of a phantom half‐filled with water. A plug‐in was written for ImageJ software for automated detection of a water surface in an image and measurement of its angle relative to a horizontal line. A water phantom can be used for the evaluation of image rotation for axial mode. In helical mode the movement of the table would cause movement of the water. The difference between image rotation for axial and helical scans can be evaluated by measuring and comparing the angles of the tabletop surface for both modes. Preliminary results of measurements for three CT scanners show that image rotation does not exceed 0.5°, and is less than 0.1° for the dedicated CT simulator. It was observed that, for one CT scanner image, the rotation angle depended on tube rotation time.

PACS number(s): 87.57.Q‐, 87.55.Qr, 06.30.Bp

## I. INTRODUCTION

Quality control in X‐ray computed tomography (CT) is generally well established. A range of tests, methods, and tolerances is given in reports and guidelines published by competent bodies, such as the AAPM, ACR, and IAEA.^1^, ^2^, ^3^ Nevertheless, several new methods of testing CT scanners have been recently discussed.^4^, ^5^, ^6^, ^7^ These include testing of parameters which have not been routinely controlled before, such as gantry rotation time,^(4)^ table feed speed,^(6)^ and gantry rotation overrun.^(7)^ A new method for measurement of half‐value layer has been also proposed.^(5)^The scope of required tests may depend on the application of the CT scanner.^(3)^ The geometric accuracy of CT images needs to be checked more thoroughly when they are used for planning radiation therapy with external beams. The tests may include checking the accuracy of distances in the image, couch movement, gantry tilt, and the level of the tabletop.^3^, ^8^ If the right and left edges of the tabletop are at different heights in a CT image, two possible causes (or a combination of both) should be considered: either the tabletop itself is not level, or the images are rotated. During CT scanning, the X‐ray tube and detectors rotate within the gantry, and data from the detectors (projections) are acquired for different angles. The projections are then used during reconstruction to create the CT image. If, during data acquisition, the registered angles of each projection differ from the physical angles, the reconstructed image may be rotated. The need to test image rotation for cone‐beam CT (CBCT) systems used in radiotherapy has already been discussed by Ayan et al.^(9)^

In this study, a method of quantitative testing of image rotation in a CT, based on automated analysis of the image of the water surface, is presented.

## II. MATERIALS AND METHODS

### A. ImageJ plug‐in for analysis of images

**Figure 1:**
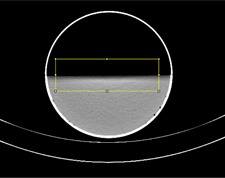
CT image of a phantom half‐filled with water displayed using ImageJ software. The user manually selects the rectangular region of interest (ROI, seen as a yellow rectangle), including the water surface.

**Figure 2:**
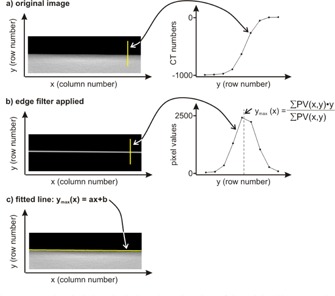
Once the “angle” ImageJ plug‐in is invoked, the selected region of the original image (a) is processed with an edge filter, the water surface is located with subpixel accuracy (b), and a straight line is fitted to the coordinates of the edge and shown as line ROI (c). The slope of the line is used to calculate the angle.

Freely available ImageJ software was used for the analysis of water surface images.^(10)^ A plug‐in named “angle” was written for automated detection of a water surface in an image and the measurement of its angle relative to a horizontal line. (The plug‐in code is provided for use by the medical physics community: Angle Plug‐in for ImageJ. Supplementary Materials are available at www.jacmp.org) Once a CT image is opened in ImageJ, the user is required to mark the water surface manually with a rectangular region of interest (ROI) (Fig. 1). After invocation of the plug‐in, the ROI is processed with the “Find Edges” filter, a built‐in feature of ImageJ (Fig. 2). The filter is based on a Sobel operator. Two convolution kernels are used to generate derivatives of pixel values in two directions: horizontal and vertical. In the processed image, the value of a pixel at any location in the image is equal to the square root of the sum of the squares of the two derivatives at that location. The obtained image of the edge is then processed column by column to find the edge with subpixel accuracy. The exact location of the edge is described as a set of coordinates (*x*,$y_{\max}$) where *x* is the number of column and $y_{\max}$(*x*) is the location of the edge in that column, calculated using the centroid method:

(1) $$y_{\max}(x)=\frac{\sum_{y}PV(x,y)\cdot y}{\sum_{y}PV(x,y)}$$

where *PV(x,y)* is the pixel value. A straight line is then fitted to the coordinates of the edge and shown in the image as line ROI. The line slope is used to calculate the angle displayed to the user.

### B. Measurements in axial mode

**Figure 3:**
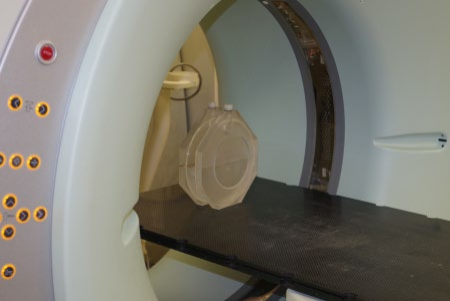
To check the image rotation angle for axial scans, a phantom half‐filled with water is positioned on the tabletop with the center of the phantom close to the isocenter of the CT scanner.

The rotation of images was measured for three CT scanners used for planning radiation therapy: a Siemens Somatom Sensation Open (wide‐bore multislice CT simulator) (Siemens Healthcare, Erlagen, German), a GE HiSpeed (single‐slice CT, also used for diagnostic examinations) (GE Healthcare, Waukesha, WI), and a Philips Gemini TF 16 (PET‐CT scanner) (Philips Medical Systems, Andover, MA). The cylindrical phantom, 20 cm in diameter, used in the measurements was originally part of a Siemens QC phantom. The phantom was half‐filled with water and positioned on a patient table using positioning lights so that the center of the phantom was close to the isocenter of the CT scanner (Fig. 3). A series of three or more CT images was obtained in axial mode with a stationary table (table feed was set to zero). Imaging was repeated for each scanner and for each available tube rotation time.

### C. Measurements in helical mode

Most examinations in our center are performed in helical mode. Since the data acquisition process differs between axial and helical scanning, it was necessary to check image rotation for helical mode as well. It was obvious that movement of the table during helical imaging would cause movement of the water surface, so the water surface could not be used as a reference. Instead, the tabletop, with no phantom, was scanned in both axial and helical modes, and the angles of the tabletop surface for both sets of images were compared. The average difference between the angles of the tabletop surface measured for images obtained in axial and helical modes at the same table position was adopted as a measure of the difference between image rotation angles in the two modes.

## III. RESULTS

## A. Repeatability of the analysis for a single image

**Figure 4:**
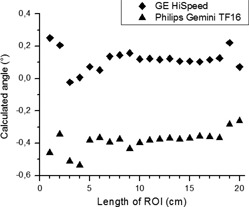
Dependence of the calculated angle of the water surface on the length of the ROI for two CT scanners.

Results for repeated analysis of the same image and the same ROI were identical. When the ROI was marked manually several times, its location and dimensions were different each time. In such a case, the range of results for repeated analysis of the same image was less than 0.05°, provided that the ROI was approximately centered in the image and its length was between 10 cm and 18 cm (Fig. 4). For ROIs shorter than 10 cm, the range of results for different ROIs was 0.3°. Due to the size of the phantom (20 cm diameter), it was hard to place a ROI larger than 18 cm so that it would not touch the phantom's walls. For subsequent measurements, a rectangular ROI 5 cm high and 16 cm wide was used. An angle of 0.05° was adopted as the threshold of detectability of image rotation.

### B. Image rotation in axial mode

For a given CT scanner and tube rotation time, the angles measured for the series of images obtained in axial mode were consistent (the range of results was less than 0.05°). For the CT simulator, practically no rotation was detected for images obtained in axial mode. An image rotation angle of up to 0.5° was observed for the PET‐CT scanner (Table 1). Interestingly, the angle was dependent on tube rotation time (+0.50° for 1.5 s compared to ‐0.36° for shorter times).

**Table 1:** Calculated angle of the water level for three CT scanners operating in axial al mode

	*Tube Rotation Time (s)*					
	*0.5*	*0.75*	*1*	*1.5*	*2*	*3*
*CT Scanner*	*Measured Angle of Water Leve*l (°)					
Siemens Somatom Sensation Open	‐0.02		‐0.01			
GE HiSpeed			0.10	0.10	0.08	0.09
Philips Gemini TF16 (PET‐CT)	‐0.36	‐0.36	‐0.35	0.50

Each result is an average of a series of at least three images. The range of results in each series was less than 0.05°.

### C. Image rotation in helical mode

**Figure 5:**
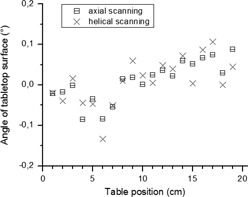
Calculated angle of the tabletop surface for the Siemens Somatom Sensation Open CT scanner for a series of axial scans and helical scans.

Differences of up to ±0.05° were observed between the tabletop surface angles measured at the same longitudinal table position in axial and helical modes (Fig. 5). Greater differences between results obtained at different table positions were observed, which might be attributable to the nonuniform structure of the tabletop. The average difference in the angles of the tabletop surface measured for axial and helical modes at the same longitudinal table position was less than 0.01°, so it was assumed that there was no difference between image rotation in axial and helical modes for that scanner and tube rotation time.

## IV. DISCUSSION

Water level can be used to check image rotation in axial mode. Unfortunately, the water surface moves along with table movement and, therefore, the method cannot be used for helical scans. A tabletop without a phantom can be used instead for measurements in helical mode. The tabletop is leveled with limited accuracy, so it cannot be used as an absolute reference. Instead, the tabletop can be scanned both in axial and helical modes, and the angles of its surface can be measured in the two modes and compared. The results of measurements with the water surface in axial mode, together with a tabletop‐based comparison of image rotation in axial and helical modes, can then be used to deduce image rotation in helical mode.

The plug‐in works if a well‐defined surface is present in the image. It works well with images of water surfaces; however, there is no guarantee that it would work well for images of all types of CT tabletops. In case of problems with the analysis of tabletop images, any flat layer of uniform material (e.g., PMMA) could be placed on the tabletop.

Ayan et al.^(9)^ suggested that in the case of CBCT used for target localization in image‐guided radiation therapy, a criterion of 1° would be a reasonable action level for image rotation. Similarly, 1° is the tolerance for gantry angle indicator accuracy for medical accelerators used for radiation therapy.^(11)^ Assuming 50 cm to be the typical width of a tabletop, image rotation of 1° would result in 9 mm of difference between the vertical position of the left and right edges of the tabletop in the image. This would be unacceptable, since, according to AAPM recommendations, in radiotherapy applications the tabletop should be level with respect to the image within ±2 mm over its width.^(8)^ If the tabletop were aligned horizontally with a precise level, any rotation between the tabletop and image would be caused by image rotation. The AAPM requirement would then be fulfilled, as long as the image was not rotated by more than

0.2°. Considering the presented results, this level of required accuracy is achievable for current CT scanners.

The described test for image rotation could also be applied to CBCT systems. The method is easy and relatively fast, although the image must be exported from the CT scanner to a workstation with ImageJ.

## V. CONCLUSIONS

Tests with water surfaces can be used for the evaluation of image rotation for axial mode. The difference between image rotation for axial and helical scans can be evaluated by replacing the water phantom with any flat surface, such as the tabletop. The preliminary results of measurements for three CT scanners show that image rotation does not exceed 0.5°, and for the dedicated CT simulator is less than 0.1°. It was observed that image rotation for a particular CT scanner might depend on tube rotation time.

## COPYRIGHT

This work is licensed under a Creative Commons Attribution 3.0 Unported License.

## Supporting information

Supplementary Material

Supplementary Material

Supplementary Material

Supplementary Material

Supplementary Material

Supplementary Material

Supplementary Material

## References

[1] American Association of Physicists in Medicine . Quality control in diagnostic radiology. AAPM Report 74. Madison, WI: Medical Physics Publishing; July 2002.

[2] American College of Radiology . Computed tomography quality control manual. Reston, VA: ACR; 2012.

[3] International Atomic Energy Agency . Quality assurance programme for computed tomography: diagnostic and therapy applications. IAEA Human Health Series 19. Vienna: IAEA; 2012.

[4] Fukuda A , Lin P , Matsubara K , Miyati T . Measurement of gantry rotation time in modern CT. J Appl Clin Med Phys. 2014;15(1):303–08. Accessed 7 September 2015. Available from: http://www.jacmp.org/index.php/jacmp/article/view/4517/html_18 10.1120/jacmp.v15i1.4517 PMC5711247 24423850

[5] Matsubara K , Ichikawa K , Murasaki Y , Hirosawa A , Koshida K . Accuracy of measuring half‐ and quarter‐value layers and appropriate aperture width of a convenient method using a lead‐covered case in X‐ray computed tomography. J Appl Clin Med Phys. 2014;15(1):309–316. Accessed 7 September 2015. Available from: http://www.jacmp.org/index.php/jacmp/article/view/4602/html_19 10.1120/jacmp.v15i1.4602 PMC5711220 24423861

[6] Fukuda A , Lin P , Matsubara K , Miyati T . Measurement of table feed speed in modern CT. J Appl Clin Med Phys. 2014;15(3):275–281. Accessed 7 September 2015. Available from: http://www.jacmp.org/index.php/jacmp/article/view/4703/html_95 24892343 10.1120/jacmp.v15i3.4703 PMC5711061

[7] Fukuda A , Lin P , Matsubara K , Miyati T . Evaluation of gantry rotation overrun in axial CT scanning. J Appl Clin Med Phys. 2014;15(5):229–234. Accessed 7 September 2015. Available from: http://www.jacmp.org/index.php/jacmp/article/view/4901/html_161 25207576 10.1120/jacmp.v15i5.4901 PMC5711073

[8] Mutic S , Palta JR , Butker EK et al. Quality assurance for computed‐tomography simulators and the computed‐tomography‐simulation process: report of the AAPM Radiation Therapy Committee Task Group No. 66. Med Phys. 2003;30(10):2762–92. 14596315 10.1118/1.1609271

[9] Ayan AS , Lin H , Yeager C et al. Should image rotation be addressed during routine cone‐beam CT quality assurance? Phys Med Biol. 2013;58(4):1059–73. 23363650 10.1088/0031-9155/58/4/1059

[10] Schneider CA , Rasband WS , Eliceiri KW . NIH Image to ImageJ: 25 years of image analysis. Nat Methods. 2012;9(7):671–75. 22930834 10.1038/nmeth.2089 PMC5554542

[11] Klein EE , Hanley J , Bayouth J et al. Task Group 142 report: quality assurance of medical accelerators. Med Phys. 2009;36(9):4197–212. 19810494 10.1118/1.3190392

