# Supplementary figures and images for: Measurement of image rotation angle in CT for radiotherapy treatment planning
Source: J Appl Clin Med Phys. 2016 Jul 8;17(4):285–90. doi: 10.1120/jacmp.v17i4.6203 (PMC5690054; doi:10.1120/jacmp.v17i4.6203)

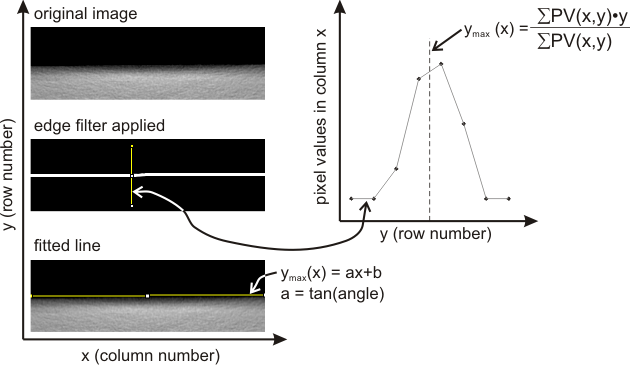

Supplement: Supplementary file 1 — Supplementary Material [file ACM2-17-285-s001.png]

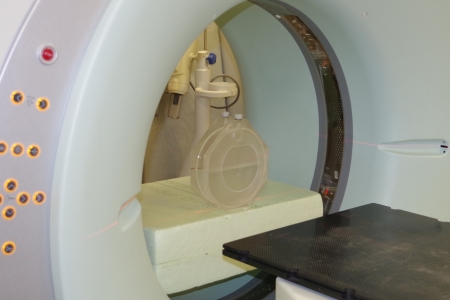

Supplement: Supplementary file 2 — Supplementary Material [file ACM2-17-285-s002.jpg]

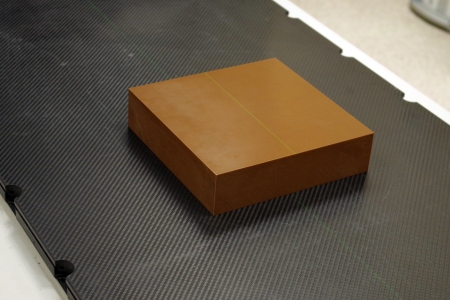

Supplement: Supplementary file 3 — Supplementary Material [file ACM2-17-285-s003.jpg]

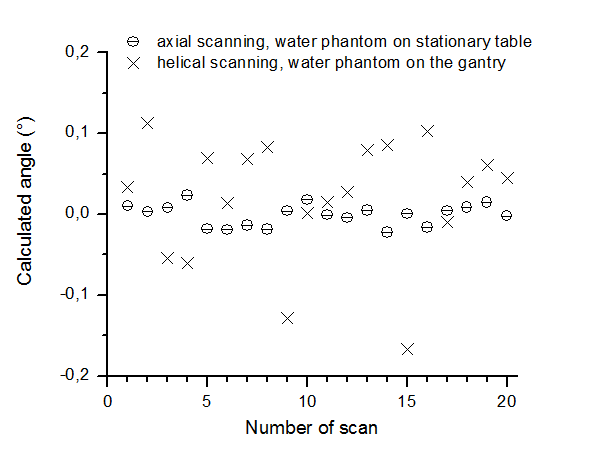

Supplement: Supplementary file 4 — Supplementary Material [file ACM2-17-285-s004.png]

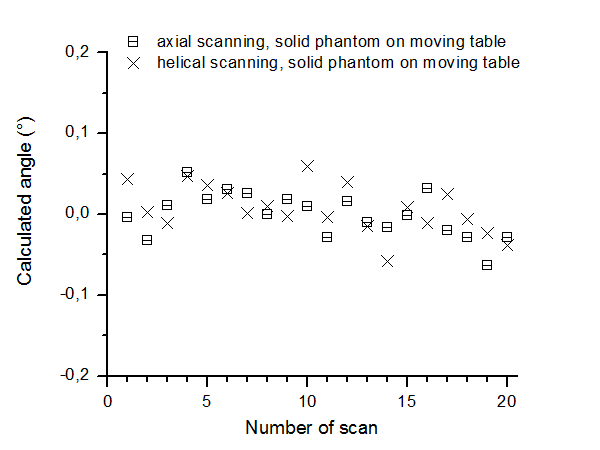

Supplement: Supplementary file 5 — Supplementary Material [file ACM2-17-285-s005.png]
